# Enhanced detection of atrial tachyarrhythmias with pacing devices by using more accurate atrial sensing

**DOI:** 10.1007/s10840-021-01066-z

**Published:** 2021-10-01

**Authors:** Sami Pakarinen, Mika Lehto, Jaap Ruiter, Willem G. de Voogt

**Affiliations:** 1 Department of Cardiology, Heart and Lung Center, Helsinki University Hospital, Haartmaninkatu 4, 00290 Helsinki, Finland; 2 Medical Center Alkmaar, Alkmaar, The Netherlands; 3 St. Lucas Andreas Hospital, Amsterdam, The Netherlands

**Keywords:** Pacing, Far-field, Atrial tachyarrhythmias, Pacemaker leads

## Abstract

**Purpose:**

Cardiac pacing devices can detect and monitor atrial tachyarrhythmias (ATA) which increase the risk of thromboembolic complications. The aim of this study was to compare (1) two different atrial leads and (2) standard and optimized settings to detect ATA and reject far-field R-wave signal (FFRW).

**Methods:**

This was a prospective, randomized multi-center trial comparing St. Jude Medical OptiSense lead (tip-to-ring spacing 1.1 mm) and Tendril lead (tip-to-ring spacing 10.0 mm), having programmed atrial sensitivity at 0.2 mV and post-ventricular atrial blanking at 60 ms. We measured intra-atrial amplitudes of FFRW, intrinsic atrial signals, the amount of FFRW oversensing, and other inappropriate mode switching.

**Results:**

One hundred and ten patients were enrolled. The mean amplitude of sensed and paced FFRW bipolar signal was 0.13 mV vs. 0.21 mV (*p* < 0.001) and 0.13 mV vs. 0.26 mV (*p* < 0.001) with OptiSense and Tendril lead, respectively. The mean amplitude of the atrial bipolar signal was 2.84 mV with OptiSense and 3.48 mV with Tendril lead, *p* = 0.014. With the optimized settings with OptiSense lead, one patient out of 20 (5%) had FFRW oversensing, none had undersensing of ATAs due to 2:1-blanking of atrial depolarizations, and the concordance of the ATAs by Holter and pacemaker memory was high (Spearman’s rank correlation coefficient = 0.90). In the Tendril group, 12 out of 25 patients (48%) had oversensing and 4 had atrial undersensing (*p* < 0.001).

**Conclusions:**

The technique with an atrial lead with short tip-to-ring spacing combined with optimized pacemaker programming resulted in reliable and accurate atrial arrhythmia detection.

**Trial registration:**

ClinicalTrials.gov number NCT01074749.

## Introduction

Pacing device diagnostics has become an important means of detecting and monitoring different types of atrial tachyarrhythmias (ATAs) including atrial fibrillation (AF), atrial flutter (AFlu), and atrial tachycardia (AT). Accurate identification of ATA as atrial high-rate episodes (AHREs) with a cardiac implantable electronic device (CIED) allows guiding of optimal anticoagulation and antiarrhythmic therapy. When therapy is guided by device memory-derived data, AHREs have to be a reliable marker for identification of onset and offset as well as incidence and duration of true ATAs. The reliability of AHRE detection depends on adequate sensing and discrimination of atrial potentials. [1–3]

Intermittent undersensing of continuous AF and undersensing of very short episodes of AF or other ATAs are often observed and may cause inappropriate AHRE detection. [1, 3, 4] And conversely, oversensing of far-field R-wave (FFRW) [5] or sensing of retrograde atrial depolarizations [4, 6, 7] may erroneously trigger AHRE detection. The problems of under- and over-sensing of atrial and ventricular signals have been studied since the early 80ties. [8]

Up to 26% of the patients with standard programming of CIED show incorrect AHRE detection due to FFRW oversensing. [5] When FFRW sensing is reduced by a less sensitive setting in the atrium or prolongation of post-ventricular atrial blanking (PVAB), AF undersensing might be expected. AFlu and AT can also be completely or intermittently ignored when blanking of every second atrial depolarization occurs (2:1-lock-in phenomenon). FFRWs and measures to avoid the result in inappropriate ATA detection and undermine the accuracy of detecting ATAs by CIED. [9]

Pacing lead characteristics have proved to be a determining factor regarding the sensing of FFRW.

Shorter spacing between the dipole—tip-to-ring—of a bipolar lead makes the pacing system less susceptible to FFRW sensing. [2, 10–15] Whether reduced FFRW sensing results in more appropriate diagnostics and therapy through increased accuracy of atrial arrhythmia detection has not yet been shown.

Silent device-detected AHREs have been associated with increased risk of stroke, mortality, and burden of AF. [16–18] Among pacemaker recipients without a history of AF, 35–72% of all strokes or systemic embolisms were preceded by AHRE detected by CIED. [16–18] Notably, with the majority of the patients’ proximate temporal relationship between device-detected AHREs and the occurrence of strokes has not been shown. [16–21] However, in these studies, the settings for detection and duration of AHREs were not optimized. Especially briefer episodes of AHRE were not included and occasional undersensing of AHREs were frequent. Accurate identification of ATA would allow appropriate patient selection for stroke prevention with anticoagulation therapy.

The aim of the OSAT’s (The Optimal Sensing in Atrial Tachyarrhythmia) study was to compare two different atrial leads in their capabilities to detect episodes and duration of paroxysmal AF and other ATAs, and rejection of FFRW oversensing with maximal sensitivity settings. The objective was also to observe and determinate if these settings would be appropriate for both of the tested leads regardless of their different designs and their ability to detect ATA and FFRW.

## Methods

### Study design

The study was a prospective, single-blinded, 1:1 randomized multicenter study. Patients were included if they had a standard indication for class I or II pacing indications for sick sinus syndrome with documented paroxysmal atrial tachyarrhythmias over the last 6 months. The exclusions were angina pectoris class ≥ III (CCS classification); symptomatic congestive heart failure—NYHA class ≥ III; severe valvular heart disease, left ventricular ejection fraction < 35%; and hypertrophic cardiomyopathy (echocardiogram less than 6 months old). The study was approved by relevant medical ethical committees and conducted according to the Declaration of Helsinki. Written informed consent was obtained for each patient prior to enrollment. The patients were treated by the decision of the doctor in charge, and the study protocol did not guide the management of arrhythmias or other clinical situations.

Patients were randomized prior implant in a 1:1 ratio to receive either OptiSense™ (model 1699 T, 1699TC, 1999 St. Jude Medical) pacing lead or Tendril™ (model ST1788T, 1788TC, ST1888TC St. Jude Medical) pacing lead in the right atrium. OptiSense lead has an inter-electrode spacing of 1.1 mm, whereas Tendril lead has an inter-electrode spacing of 10 mm. The lead characteristics have been described in detail elsewhere. [11–15] Both groups of patients were subjected to the same implantation protocol and follow-up schedule.

### Implantation and measurements

A conventional right ventricular (RV) lead was positioned in the RV septum or RV apex depending on the preference of implanting physician. Thereafter, the short tip-to-ring or conventional lead was placed to standard position into the right atrial appendage (RAA) and if not anatomically possible then to another location. The lead location was confirmed by fluoroscopy.

All patients received the St. Jude Medical Accent™ pacemaker (model PM2112/PM2212), capable of storing atrial electrograms (EGMs) starting 10 s before and lasting 10 s after automatic mode switch (AMS) initiation triggered by AHRE. The maximum storage of the device is up to 14 min which results in a maximum of 21 mode switch episodes with stored EGMs. The number and duration of AMS episodes as well as the total amount of AMS are also recorded by the device. The AMS algorithm has been described more in detail previously. [22–24]

The following measurements were recorded at implant and pre-hospital discharge: atrial signal amplitude; capture threshold and lead impedance with bipolar pacing with a pulse width of 0.4 ms and 5.0 Volt/0.4 ms, respectively; and intrinsic FFRW amplitude and paced FFRW amplitude. A standard St. Jude Medical Merlin™ programmer was used for the measurements. Atrial signal and FFRW amplitudes were measured manually from programmer print-outs. The amplitude of the FFRW signal was measured from peak-to-peak and recorded during intrinsic sinus rhythm (referred to as intrinsic FFRW) and during right ventricular pacing (paced FFRW). Measurements with an unstable baseline or during atrial fibrillation were excluded from the analysis. Patients were discharged the day after implantation after confirmation of the lead position by standard chest radiography.

### Holter and pacemaker settings

At the follow-up visits, between 1 and 3 months after implantation, patients had a 2-channel Holter recorded over 3-day and 4-day periods, respectively (Lifescreen, Del Mar Reynolds Medical, Hertford, UK). During the first 3-day Holter recording pacemaker settings were sensitivity of 0.2 mV in atrium and PVAB of 60 ms (“OSAT-settings”). The atrial tachycardia detection rate was set to 180 bpm. The timing of the Holter monitor and pacemaker was synchronized by the application of a magnet on the pacemaker, resulting in DOO pacing that was readily identified at the start of the Holter recordings. Analysis was performed as the patient was blinded and both automatically and visually controlled by an independent-blinded core laboratory. After the 3-day Holter recording patients were evaluated through their PM diagnostic data. Before starting the 4-day Holter recording period, the need for changing pacemaker settings from the OSAT settings to individually selected, optimized settings was considered by the discretion of the treating study physician if an occasional FFRW was seen. After the 4-day Holter recording, the follow-ups were completed.

Holter recordings were assessed by the core lab with regard to the total duration and number of episodes of ATAs. From the pacemaker, memory episodes of mode switching were compared to the Holter assessment. EGMs for inappropriate mode switching episodes were analyzed. Results were divided into two categories: no inappropriate mode switching and inappropriate mode switching due to FFRW oversensing and/or undersensing of ATAs due to 2:1-blanking of atrial depolarizations. EGM strips were not available for all AMS episodes. In this case, the summary of the episodes was used to determine the number and duration of AMS episodes.

Prior to the Holter recording periods, a myopotential sensing test was performed during both of the follow-ups, consisting of two 15 s continuous provocation tests. During the test, a continuous EGM and surface ECG recording was recorded through the pacemaker programmer.

### Statistical analyses

Continuous variables are expressed as means ± standard deviation. All pacemaker-recorded AF episodes throughout the study were verified by reviewers to identify FFRW sensing and/or artifacts or undersensing recordings. Between-group comparisons were made by Mann–Whitney’s *U* test for continuous variables and by Fisher’s exact test for contingency. The correlation between ATA burden and mode switches as measured by Holter and pacemaker was evaluated with Spearman’s rank correlation coefficient. A *p* value < 0.05 was considered statistically significant.

## Results

### Patient characteristics

One hundred ten patients were randomized in the study at 12 clinical centers. Fourteen patients did not complete the 7-day follow-up scheme due to several reasons, comprising no ATAs, permanent AF, lost to follow-up, and patient withdrawal. Patient demographics and distribution between the randomization groups are presented in Table 1. Atrial lead was implanted mainly (85%) in the appendage, and ventricular lead was implanted most often (63%) in the apical position.

**Table 1:** Characteristics of the randomized patients and lead position data

	Tendril	OptiSense	*P* value
Gender			0.96
Female	28	33	
Male	24	25	
All	52	58	
Age	73 ± 9	73 ± 10	0.76
Lead position atrial lead			0.16
Appendage	46	48	
Septal High	2	0	
Septal Low	0	1	
Free wall mid	2	4	
Free wall High	2	1	
Other	0	4	
All	52	58	
Lead position ventricular lead			0.53
Apical	31	38	
RVOT	2	0	
Septal	18	18	
Other	1	2	
All	52	58	
Echo data			
LA (mm)	41 ± 7	42 ± 8	0.71
LVEDD (mm)	46 ± 7	46 ± 10	0.98
LVEF (%)	58 ± 11	58 ± 10	0.94
Cardiovascular history			
Revascularization (PCI/CABG)	11	10	
Valvular heart disease	1	8	
Hypertension	30	28	
Coronary artery disease	9	10	
Myocardial infarction	8	7	
Other	12	22	

*RVOT* right ventricle outflow tract, *LA* left atrium, *LVEDD* left ventricle end-diastolic diameter, *LVEF* left ventricle ejection fraction

The mean amplitude of sensed and paced FFRW bipolar signal was lower with OptiSense lead compared to Tendril lead 0.13 mV vs. 0.21 mV (*p* < 0.001) and 0.13 mV vs. 0.26 mV (*p* < 0.001), respectively (Table 2). The amplitude of the atrial bipolar signal was lower with OptiSense lead compared to Tendril lead, 2.84 mV vs. 3.48 mV, *p* = 0.014. The unipolar atrial sense signal did not differ between both groups. There were no differences in atrial threshold or impedance measurements between the groups.

**Table 2:** Pacemaker measurement in the Tendril and OptiSense lead groups

	Tendril	OptiSense	*P* value
Atrial unipolar signal (mV)	3.32 ± 1.46	3.35 ± 1.62	ns
Atrial bipolar signal (mV)	3.48 ± 1.39	2.84 ± 1.32	0.0144
Atrial threshold (V)	0.71 ± 0.3	0.75 ± 0.6	ns
Atrial Impedance (Ohms)	442 ± 73	434 ± 80	ns
FFRW sensed unipolar (mV)	1.94 ± 0.89	2.11 ± 1.62	ns
FFRW paced unipolar (mV)	1.36 ± 0.75	1.48 ± 1.42	ns
FFRW sensed bipolar (mV)	0.21 ± 0.13	0.13 ± 0.24	< 0.001
FFRW paced bipolar (mV)	0.26 ± 0.15	0.13 ± 0.18	< 0.001

**Fig. 1:**
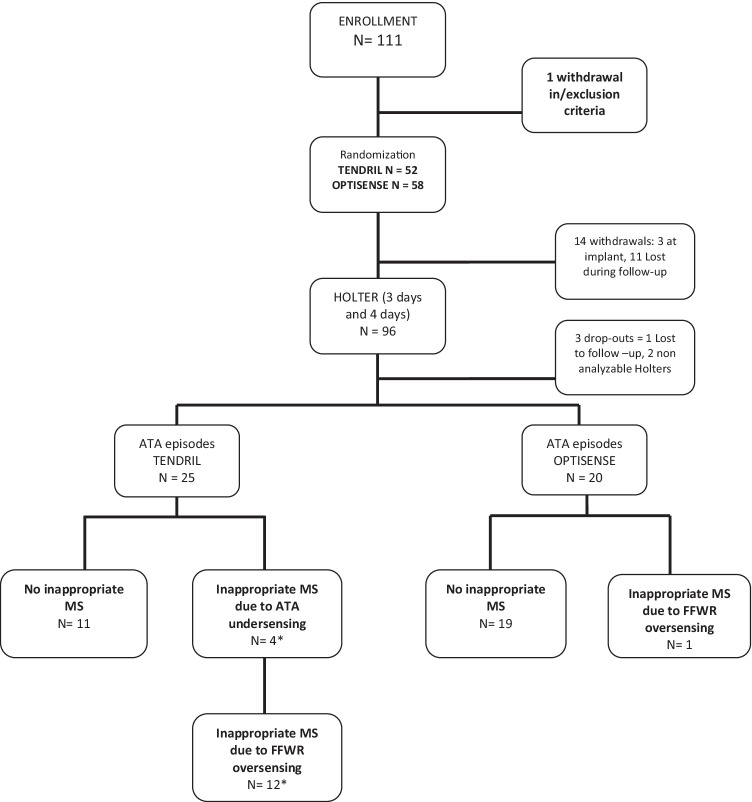
Study flow chart and evaluation of the AMS detections. * Two patients had both ATA undersensing and FFRW oversensing. MS = mode switch, FFRW = far-field R-wave

For 96 patients, 3- and 4-day Holter recordings were performed and reviewed for ATAs. Ninety-three patients had sufficient interpretable time for the analysis. ATA’s and/or AMS episodes were seen in 20 patients with the OptiSense lead and in 25 with the Tendril lead (Fig. 1). Fourteen patients out of those 45 had ATA runs in their Holter recordings in both lead groups. Table 3 shows statistically significant correlations between the total time in AMS recorded by the pacemaker and Holter-verified ATA duration for both groups, after 3 and 4 additional days of Holter. On the contrary, no statistically significant correlation in the total number of ATA episodes between the Holter and pacemaker recordings (*p* > 0.05) in either of the groups was found (Table 4).

**Table 3:** Correlation between the total time in AMS calculated by the pacemaker and the Holter-detected ATA duration in patients with OptiSense and Tendril leads

Holter	OptiSense lead		Correlation	Tendril lead		Correlation
	PM AMS duration (minute)	Holter ATA duration (minute)		PM AMS duration (minute)	Holter ATA duration (minute)	
Device programmed with maximal sensitivity settings	14,533	14,957	0.90 (*p* < 0.001)	14,813	7860	0.36 (*p* = 0.003)
Device programmed with individualized sensitivity settings	22,970	20,712	0.54 (*p* < 0.001)	20,814	21,022	0.68 (*p* < 0.001)

**Table 4:** Correlation between the number of AMS episodes in the pacemaker and number of AT/AF episodes in the Holter in patients with OptiSense and Tendril leads

Holter	OptiSense lead		Correlation	Tendril lead		Correlation
	PM AMS numbers	Holter ATA numbers		PM AMS numbers	Holter ATA numbers	
Device programmed with maximal sensitivity settings	1310	506	0.17 *p* = 0.116	20,715	1092	0.009 *p* > 0.05
Device programmed with individualized sensitivity settings	1367	415	0.013 *p* > 0.05	7860	1079	0.03 *p* > 0.05

With the OSAT settings, patients with OptiSense lead only one patient out of 20 (5%) had FFRW occurrence and no 2:1-blanking was seen. In the Tendril lead group, 12 out of 25 patients (48%) had inappropriate mode switching due to FFRW oversensing and 4 had undersensing of ATAs due to 2:1 blanking (*p* < 0.001). Two patients had both ATA undersensing and FFRW oversensing.

**Fig. 2:**
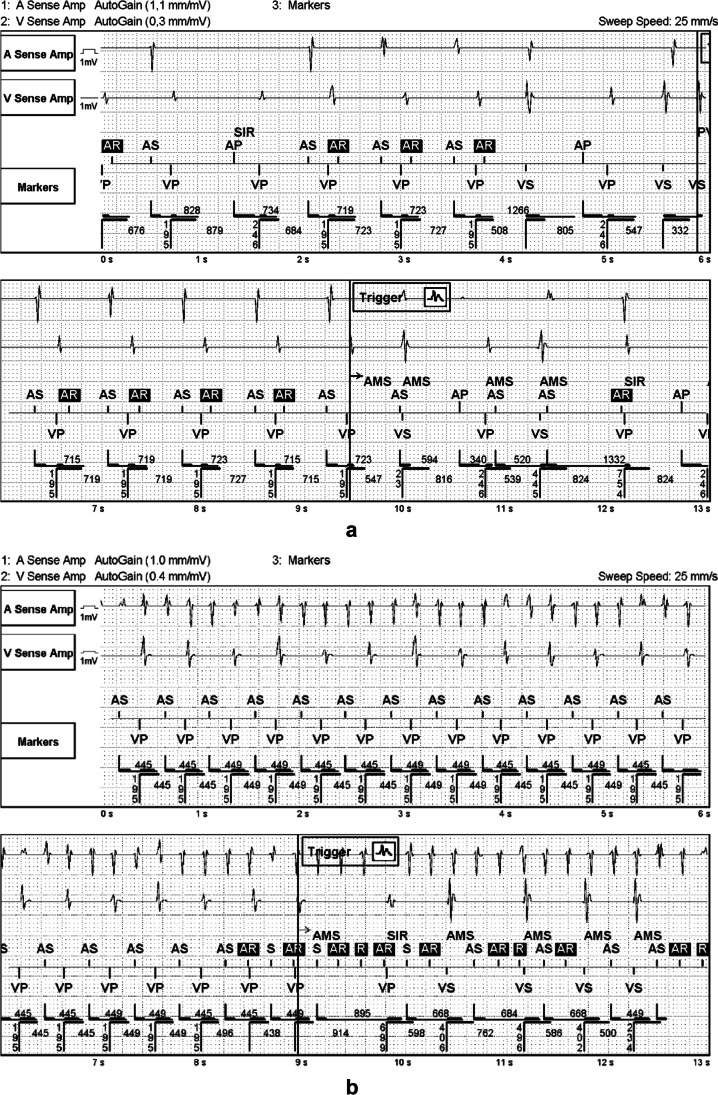
**a** Inappropriate automatic mode switch (AMS) due to far field R-wave (FFRW) signals in a patient with Tendril lead. 70 ms after the ventricular paced event (VP), a FFRW signal (AR in a box) is seen by the pacemaker and an inappropriate AMS is triggered. Post-ventricular atrial blanking period (PVAB) was previously programmed to 60 ms, and because of FFRW oversensing, it was prolonged to 140 ms at this session. **b** Undersensing of atrial flutter in **a** patient with Tendril lead, the same patient than in a with PVAB setting prolonged to 140 ms because of FFRW. The patient has an episode of atrial flutter for an undetermined time. Every second atrial signal occurs during the PVAB and therefore cannot be sensed. Eventually, due to prolongation of the paced atrioventricular interval, blanked atrial signals (AR in a box) now fall outside the PVAB and AMS is triggered

After 3 days of Holter monitoring, the treating physician had a possibility to change pacemaker settings from the OSAT settings in her/his discretion if occasional FFRW was seen. Twelve out of 25 patients in the Tendril group had modifications done to their pacemaker settings. The justification for the changes was documented as FFRW in 11 patients. For these patients, the sensitivity was increased and PVAB prolonged. In four patients who had documented FFRW and thereof their PVAB was prolonged, an undersensing of AFlu was seen at the end of the Holter period because of 2:1-blanking phenomenon. Three patients with OptiSense lead had their pacemaker settings changed: one patient had FFRW, one had occasional undersensing of AF, and one patient’s settings were changed unintentionally. Examples of undersensing of atrial flutter and of inappropriate mode switching because of FFRW are shown in Fig. 2a, b.

Sensing of myopotentials was performed at follow-ups, and no myopotential sensing was seen in the analyzed subjects in both groups.

## Discussion

The main finding of this study is that using settings with high sensitivity for ATA detection, i.e., atrial sensitivity of 0.2 mV and a short PVAB of 60 ms, and the pacing system is less susceptible to FFRW oversensing with an atrial lead with a short tip-to-ring spacing compared with atrial leads with standard tip-to-ring spacing, without a compromise on the true ATA detection. Introducing this practice (high sensitivity for ATA detection and short tip-to-ring spacing leads) results in increasingly correct and accurate atrial arrhythmia detection. To our best knowledge, OptiSense lead with these settings is a state of the art regarding diagnostics of AF with pacing devices.

This study showed that a lead with shorter tip-to-ring spacing both the amplitude of sensed and paced FFRW signal was low and was considerably lower compared to the 10-mm tip-to-ring. However, adequate atrial signal sensing amplitudes were maintained. These findings are in accordance with previous studies. [11–15]

The use of a shorter tip-to-ring spacing lead and the abovementioned optimal sensitivity settings gave a high concordance with the total duration of ATA by continuous Holter recordings and the total duration of AMS derived by the pacemaker diagnostics. Only one patient had inappropriate AMS episodes due to FFRW oversensing, and after programming longer PVAB, no FFRW oversensing was seen. None of the patients with OptiSense lead and the OSAT settings had undersensing of AFlu or atrial tachycardia.

As shown in Table 3, the total duration of ATAs by Holter recording was vastly concordant (Spearman’s rank correlation coefficient = 0.90) with the total duration of AMS measured from the pacemaker diagnostics in the OptiSense group with the OSAT-settings but became worse at the fourth day of Holter recording due to inappropriate oversensing of AHREs after the settings were changed with three patients. In the Tendril group, the correlation was poorer (Spearman’s rank correlation coefficient = 0.36) with OSAT-settings and improved with individualized programming (Spearman’s rank correlation coefficient = 0.68). In patients with Tendril leads and OSAT-settings, FFRW oversensing caused inappropriate detection of AHREs in 12 subjects; this led to the change of settings which thereafter yielded to a decrease in false AHRE detection in the fourth day of Holter recording. Our interpretation of these data is that with OptiSense lead the OSAT sensitivity settings can be used, while, with conventional atrial leads, high sensitivity settings cannot be recommended to be used.

The accuracy of pacemaker measurement of ATA burden compared with the measurement performed by Holter is an important finding of our study, because it proves that device ATA diagnostics can be used to alert about increased stroke risk; indeed, several studies have shown the association between maximum daily ATA duration or total ATA burden and risk of stroke. [16–18]

The number of AMS episodes had no correlation with Holter-documented AF episodes, and the number of the AMS episodes was higher seen by the device than the Holter. This could result from that the pacemaker counter will falsely register separated periods of ATA, although continuous ATA persists, probably due to undersensing of low voltage ATA episodes (Fig. 2b). Similar findings have been shown in other studies which showed that undersensing may cause inappropriate episode detection end and that runs of premature atrial contractions may cause inappropriate episode detection start [1, 25].

Half of the patients with Tendril lead had FFRW oversensing episodes with the abovementioned optimal sensitivity settings for ATA detection. The amount of time in AMS was incorrectly almost two times higher than the total duration of ATAs in the Holter recordings. After prolongation of PVAB, four (12%) patients had undersensing of AFlu or atrial tachycardia due to 2:1-lock occurrence. Thus, it seems that the high sensitivity settings for ATA detection, while optimal for the OptiSense-lead, cannot be recommended to be used with a conventional atrial lead.

In our study, no false AHRE detection was due to repetitive non-reentrant ventriculo-atrial synchronous rhythm (RNRVAS). In the ASSERT trial, 17.3% of the AHREs were false mostly due to RNRVAS. [6] In our study, overdrive atrial pacing or longer AV-intervals were not used which can predispose to RNRVAS.

In previous studies, the proximate temporal relationship between device-detected AF and the occurrence of strokes has not been shown. [16–21] However, in these studies, pacemaker settings have been suboptimal for AF detection. Especially shorter episodes of AF were not included, and occasional undersensing of longer AF episodes were frequently limiting the ability to reveal a possible association of these shorter episodes and stroke. Also, some thromboembolic events may be due to chronic endothelial changes due to multiple short prior ATA episodes. Furthermore, the occurrence of even brief ATA episodes may trigger chronic changes in the atrium which may lead to thrombus formation sometime after the occurrence of a longer episode of ATA.

### Clinical implications

In patients wearing cardiac implantable devices, the association between device-detected atrial tachyarrhythmias and stroke or systemic thromboembolism has been convincingly shown by many studies. [16–18] At present, there is no evidence in support of or against the prescription of oral anticoagulants in patients at increased risk of stroke who present with AHREs. For this reason, two randomized controlled trials are ongoing for evaluating the efficacy and risk–benefit ratio of oral anticoagulation vs no oral anticoagulation (aspirin alone as the comparator), in patients with device-detected atrial tachyarrhythmias, respectively, the ARTESiA study [26] and NOAH/AFNET study. [27] If these studies will show the clinical value of CIED-detected AHRE, accurate detection of atrial tachyarrhythmias will be confirmed as a feature of paramount clinical importance in cardiac implantable devices specifically for the prevention of embolic complications. Accurate identification of ATA, like that obtainable with a short tip to ring distance leads, would be useful and eventually allow better patient selection for stroke prevention with antithrombotic therapy.

## Conclusions

The use of an atrial lead with a short 1.1 mm tip-to-ring spacing and high sensitivity settings resulted in reliable atrial arrhythmia detection, more accurate than using standard tip-to-ring spacing leads. The high sensitivity settings, which appear to provide accurate ATA detection in the 1.1-mm tip-ring spacing lead, cannot be recommended to be used with a conventional atrial lead.

## Appendix

OSAT-Investigators:

- M.Lehto and S. Pakarinen (Helsinki University Central Hospital, Finland)
- W.G. de Voogt (St. Lucas Andreas Hospital, the Netherlands)
- G.S. de Ruiter (Onze Lieve Vrouwe Gasthuis, the Netherlands)
- J.H.Ruiter (Medical Center Alkmaar, the Netherlands)
- E.Viergever (Groene Hart Hospital, the Netherlands)
- J.Voitk (North Estonia Medical Center, Estonia)
- L.H.R. Bouwels (Canisius Wilhelmina Hospital, the Netherlands)
- R. Hazeleger (VieCuri Medical Center, the Netherlands)
- Y.S. Tuininga, (Deventer Hospital, the Netherlands)
- R.M. Robles de Medina (Haga Hospital, the Netherlands)
- S.A.M. Said (Hospital Group Twente, the Netherlands)
- J.G. Engbers (Scheper Hospital, the Netherlands)
